# Hypoglycemic and antioxidant effects of leaf essential oil of *Pelargonium graveolens* L’Hér. in alloxan induced diabetic rats

**DOI:** 10.1186/1476-511X-11-81

**Published:** 2012-06-26

**Authors:** Maher Boukhris, Mohamed Bouaziz, Ines Feki, Hedya Jemai, Abdelfattah El Feki, Sami Sayadi

**Affiliations:** 1 Laboratoire des Bioprocédés Environnementaux, Centre de Biotechnologie de Sfax, Université de Sfax, BP: «1177», 3018 Sfax, Tunisia; 2 Laboratoire d’Ecophysiologie animale, Faculté des Sciences de Sfax, B.P. «802», 3018 Sfax, Tunisia

**Keywords:** *P. graveolens*, Diabetes, Glibenclamide, Alloxan, Rats, Antioxidant activity

## Abstract

**Background:**

Rose-scented geranium (*Pelargonium graveolens* L’Hér.), which is used in traditional Tunisian folk medicine for the treatment of hyperglycaemia, is widely known as one of the medicinal herbs with the highest antioxidant activity. The present paper is conducted to test the hypoglycemic and antioxidative activities of the leaf essential oil of *P. graveolens*.

**Methods:**

The essential oil *P. graveolens* was administered daily and orally to the rats at two doses of 75 mg/kg and 150 mg/kg body weight (b.w.) for 30 days. The chemical composition of *P. graveolens* essential oil, body weight, serum glucose, hepatic glycogen, thiobarbituric acid-reactive substances (TBARS), the components of hepatic, and renal and serum antioxidant systems were evaluated. The hypoglycemic effect of rose-scented geranium was compared to that of the known anti-diabetic drug glibenclamide (600 μg/kg b.w.).

**Results:**

After the administration of two doses of essential oil of *Pelargonium graveolens* L’Hér. together with glibenclamide which is known by its antidiabetic activities and used as reference (600 μg/kg b.w.), for four weeks, the serum glucose significantly decreased and antioxidant perturbations were restored. The hypoglycemic effect of *P. graveolens* at the dose of 150 mg/kg b.w. was significantly (*p*< 0.05) more effective than that of glibenclamide. It is through the histological findings in hepatic and renal tissues of diabetic rats that these beneficial effects of geranium oils were confirmed.

**Conclusions:**

It suggests that administration of essential oil of *P. graveolens* may be helpful in the prevention of diabetic complications associated with oxidative stress. Our results, therefore, suggest that the rose-scented geranium could be used as a safe alternative antihyperglycemic drug for diabetic patients.

## Background

An overview of the diabetes mellitus (DM) prevalence in Tunisia shows that it corresponds to 9.3–11.0% (between 2010 and 2030), which is in accordance with the percentage observed in other countries. The world prevalence of diabetes among adults (aged 20–79 years) is 6.4%, corresponding to 285 million, in 2010, and will be estimated by 7.7%, equivalent to 439 million, by 2030. In fact, it is one of the most common chronic diseases in nearly all countries, and continues to increase in number and significance due to the changing lifestyles that lead to the reduction of physical activity and increase of obesity [1].

Numerous studies have demonstrated that oxidative stress, mediated mainly by hyperglycemia-induced generation of free radicals, contributes to the development and progression of diabetes and its complications [2-4]. The abnormally high level of free radicals leads to membrane damage because of membrane lipids peroxidation, protein glycation and the simultaneous decline of antioxidant defense mechanisms [4,5]. Nowadays, although the major agents used for diabetes treatment are synthetic drugs and insulin, they usually come with considerable side effects, such as hypoglycemia, drug-resistance, dropsy, and weight gain [6]. However, as hundreds of traditional folk medicines have proven to be efficient for the treatment of diabetes with less tolerability and side effects, there is an increasing trend towards searching for more natural antidiabetic agents.

Rose-scented geranium is an important aromatic plant that grows in temperate areas of the world [7] including those in Tunisia. Geranium oil has a fine rosy odor with pronounced fruity minty undertone and a rich long-lasting sweet-rosy dryout [8]. As the cost of its highly valued essential oil, which is used in soaps, perfumery, and cosmetic industries, is increasing in international market, attempts were made to promote its cultivation by employing various agrotechnologies [9].

The essential oil of *P. graveolens* has been used for many years in traditional medicine as antiasthmatic, antiallergic, antioxidant, antidiarrhoeic, antihepatotoxic, diuretic, tonic, haemostatic, stomachic and diabetic [10-14].

It is worthwhile to note that no detailed studies have been carried out on the efficiency of *Pelargonium graveolens* in the modulation of oxidative stress pertaining to DM in experimental animals. Hence, the present study undertakes the investigation of possible hypoglycemic and antioxidant effects of *Pelargonium graveolens* leaves in alloxan-induced diabetic rats. Indeed, the essential oil of rose-scented geranium leaves was prepared and their antioxidant activities as well as their hypoglycemic effects were studied.

## Results

### Chemical constitution of leaf essential oil

The hydrodistillation of the leaves of *P. graveolens* generated pale yellow coloured oil (yield of 0.19%, v/w). Upon GC/MS analysis, the essential oil was found to contain 47 constituents, 96.23% of which were identified (Table 1). All constituents were eluted between 8 and 31 min. The volatile oil contained 67.39% monoterpenoids, 25.4% sesquiterpenoids, and 3.44 other compounds. The major components were β-citronellol (16.24%) followed by geraniol (15.30%), δ-selinene (8.69%), citronellyl formate (7.39%), geranyl formate (6.47%), l-menthone (4.13%) and linalool-l (3.8%). Twenty nine components of the oil amounted to <1%.

**Table 1:** **Chemical composition of leaf essential oil of*Pelargonium graveolens*L’Hér**

**N°**	**Composant**	**RT**	**KI**^**a**^	**%**
1	α-pinene	8.870	930	0.66
2	β-myrcene	10.633	997	0.18
3	phellandrene	10.010	1007	0.09
4	β-phellandrene	11.766	1023	0.21
5	cis-ocimene	12.063	1030	0.16
6	β-ocimene	12.378	1037	0.13
7	linalool l	14.003	1073	3.80
8	rose oxide-trans	14.772	1079	0.54
9	l-menthone	15.908	1141	4.13
10	3-p-menthanol	16.418	1186	0.14
11	α-terpineol	16.646	1197	0.24
12	β-citronellol	17.894	1237	16.24
13	z-citral	18.106	1244	0.71
14	nerol	18.495	1259	3.08
15	geraniol	18.706	1266	15.30
16	e-citral	18.981	1277	0.84
17	citronellyl formate	19.113	1282	7.39
18	geraniol formate	19.834	1309	6.47
19	propanoic acid	21.087	1357	0.39
20	lavandulyl acetate	21.893	1388	0.76
21	β-bourbonene	22.014	1393	1.01
22	trans-caryophyllene	22.912	1427	1.63
23	β-cubebene	23.129	1434	0.64
24	citronellyl propionate	23.393	1444	0.14
25	azulene	23.473	1449	0.40
26	α-cubebene	23.662	1458	0.96
27	α-humulene	23.759	1460	0.51
28	aromadendrene	23.942	1467	0.68
29	germacrene-d	24.457	1486	2.87
30	β-selinene	24.577	1492	0.17
31	β-cuvebene	24.709	1496	0.32
32	ledene	24.806	1500	3.10
33	α-muurolene	24.880	1504	0.18
34	elemol	25.029	1510	0.40
35	α-amorphene	25.229	1519	0.29
36	δ-cadinene	25.447	1528	1.36
37	epizonaren	25.504	1530	0.24
38	α-gurjunene	25.790	1545	0.55
39	α-agarofuran	26.030	1553	0.57
40	neryl acetate	26.236	1563	1.06
41	phenylethyl tiglate	26.894	1591	2.07
42	δ-selinene	27.816	1631	8.69
43	agarospirol	28.090	1643	0.46
44	isoledene	28.474	1660	1.18
45	propanoate	28.657	1667	0.43
46	geranyl tiglate	29.475	1703	3.05
47	mintsulfide	30.373	1746	1.81
	Total			96.23
	Monoterpenes hydrocarbons			1.43
	Oxygenated monoterpenes			65.96
	Sesquiterpenes hydrocarbons			24.54
	Oxygenated sesquiterpenes			0.86
	Other compounds			3.44

^a^ KI, retention index (Kovalts) relative to *n*-alkanes (C9–C26) on a polar DB-5 MS column.

### Body weight

Table 2 shows the body weight in normal and experimental animals in each group. Indeed, the injection of alloxan (150 mg/kg) reduced the body weight of diabetic rats compared with controls. The mean body weight of diabetic rats showed a decrease by 19.74% compared with normal animals. The body weight of diabetic rats treated with essential oil significantly increased as compared with non-treated diabetic animals.

**Table 2:** Growth Performance of the experimental Rats*

**Groups**	**Initial body wt (g)**	**Final body wt (g)**
Control	230.16 ± 5.55 ^a^	318.24 ± 9.12 ^a^
Diabetic	225.87 ± 7.94 ^a^	255.39 ± 11.89 ^b^
Glibenclamide (600 μg/kg b.w.)	227.97 ± 6.20 ^a^	277.17 ± 10.23 ^c^
EO 1 (75 mg/kg b.w.)	228.22 ± 6.32 ^a^	280.09 ± 7.66 ^c^
EO 2 (150 mg/kg b.w.)	230.11 ± 5.96 ^a^	301.83 ± 10.54 ^d^

^abcd^ The means in the row not sharing a common letter are significantly different among groups (*p* < 0.05).

* Values are expressed as means ± S.E. (n = 8).

### Serum glucose and hepatic glycogen concentration

**Figure 1:**
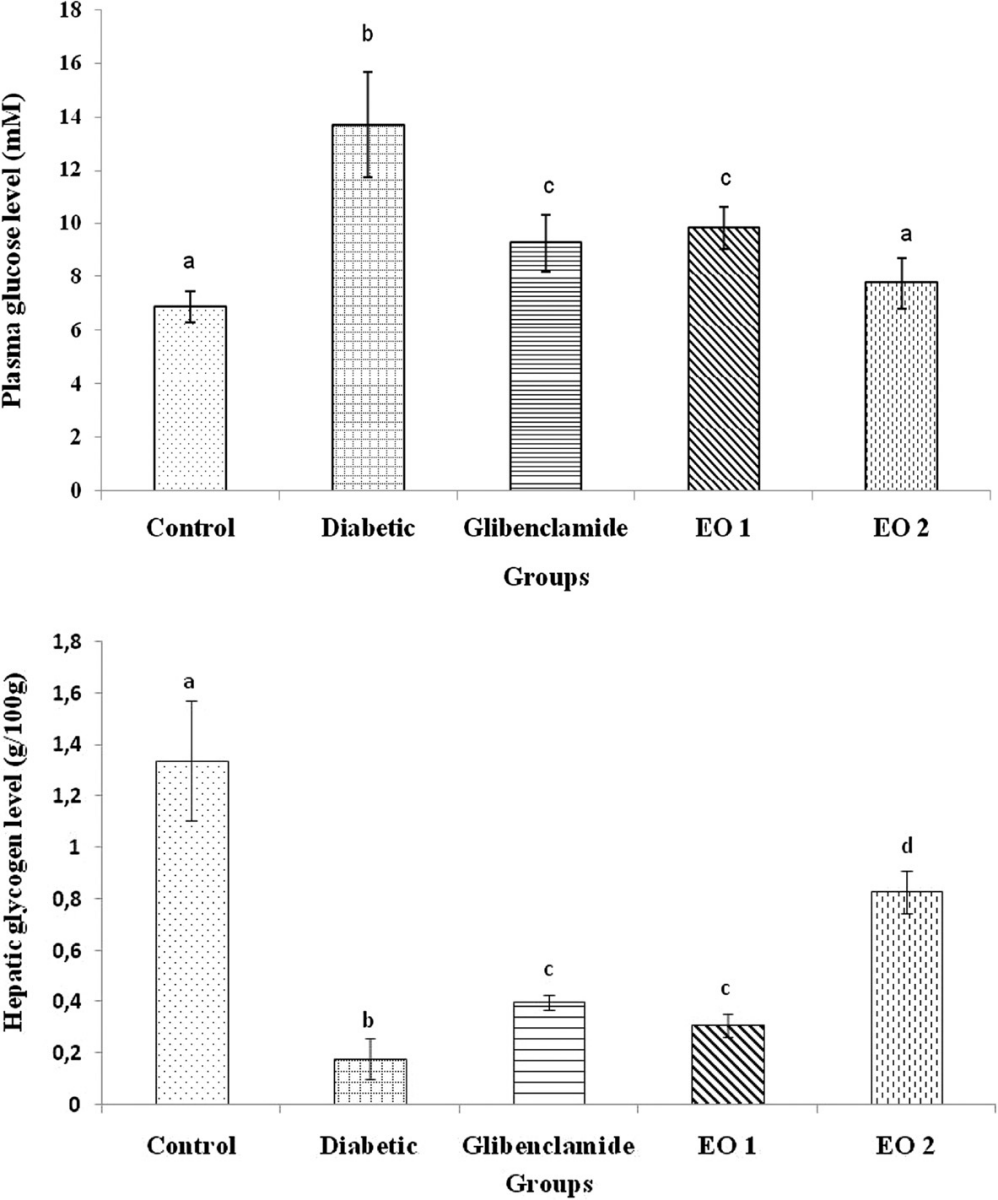
**Effects of leaf essential oil (EO1 and EO2) and glibenclamide on serum glucose levels and liver glycogen levels in normal and alloxan-induced diabetic male wistar rats for 30 days.** Values are expressed as means ± S.E. (n = 8).^abcd^ The means not sharing a common letter are significantly different among groups (*p* < 0.05).

**Figure 2:**
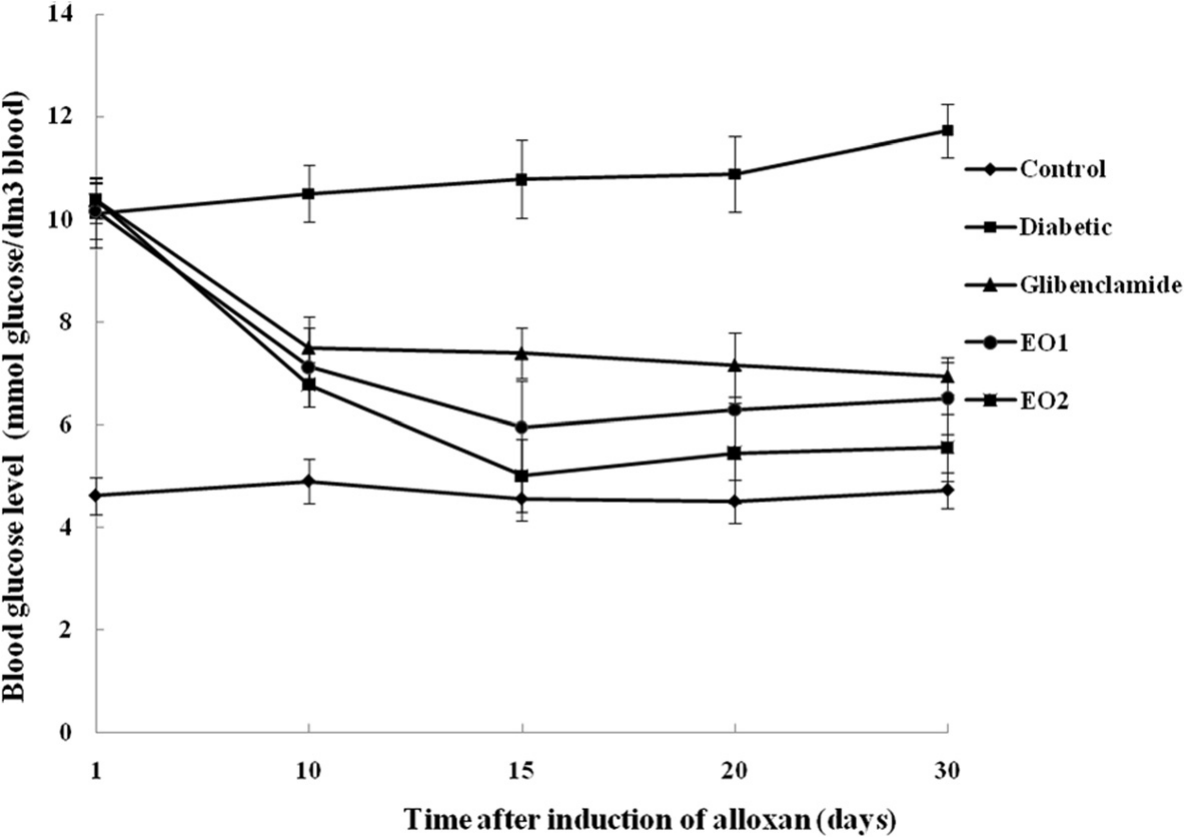
**Effects of leaf essential oil (EO1 and EO2) on blood glucose levels in normal and alloxan-induced diabetic male wistar rats for 30 days.** Values are expressed as means ± S.E. (n = 8). Values differ significantly at *p* < 0.05.

The serum glucose levels of diabetic rats were significantly higher compared with those of the control group (*p* < 0.05) (Figure 1). After the administration of the leaf essential oil of *P. graveolens*, a significant decrease in blood glucose was observed compared with that of the diabetic group (*p* < 0.05) (Figure 2). Moreover, rats receiving essential oil at 150 mg/kg b.w. illustrated a significantly pronounced antidiabetic effect compared with those receiving these compounds at 75 mg/kg b.w. However, the hepatic glycogen levels showed a significant decrease by 86.56% in diabetic rats compared with those in the controls (Figure 1). Furthermore, all of the groups receiving the two doses of essential oil of *P. graveolens* depicted a significant increase in glycogen levels compared with those of the normal and with the diabetic controls. The rats receiving essential oil at 150 mg/kg b.w. showed the significantly highest levels of hepatic glycogen concentration (*p* < 0.05).

### Hepatic function, diabetes and *P. graveolens* preparations

**Figure 3:**
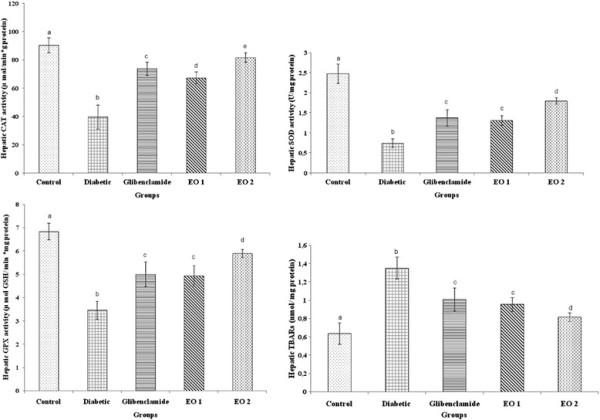
**Effects of leaf essential oil (EO1 and EO2) and Glibenclamide on hepatic SOD, CAT, GPX activities and TBARS of diabetic male wistar rats for 30 days.** Values are expressed as means ± S.E. (n = 8). ^abcd^ The means not sharing a common letter are significantly different among groups (*p* < 0.05).

**Figure 4:**
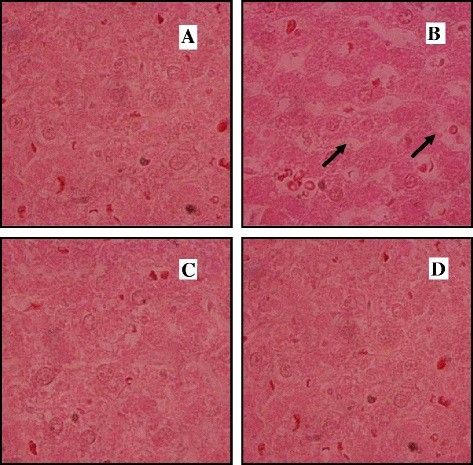
**(A) Normal rat liver.** (**B**) Diabetic rat liver: sinusoidal congestion and fatty degeneration in the form of fat lake. (**C**) Diabetic rat treated with Glibeclemide and (**D**) Diabetic rat treated with essential oil (150 mg/kg): a positive effect was observed (H&E 100×).

This study has demonstrated a decrease in the SOD (superoxide dismutase), CAT (catalase) and GPX (glutathione peroxidase) activities in hepatic tissues of diabetic rats together with an increase in plasma TBARS levels (Figure 3). In diabetic rats treated with two doses of essential oil, a clear protective effect was observed in hepatic function and metabolism. In fact, the administration of glibenclamide inhibited all changes caused by alloxan-induced diabetes. This positive effect of the essential oil was confirmed by histological findings (Figure 4). As shown in Figure 4B, vacuolated hepatocytes with the nucleus being pushed to the periphery and fatty cyst were observed in diabetic rats. This structure is different compared with the livers of normal control rats. However, the essential oil administration to diabetic rats reduced the appearance of fat cells in liver (Figure 4D).

### Renal function, diabetes and *P. graveolens* preparations

**Figure 5:**
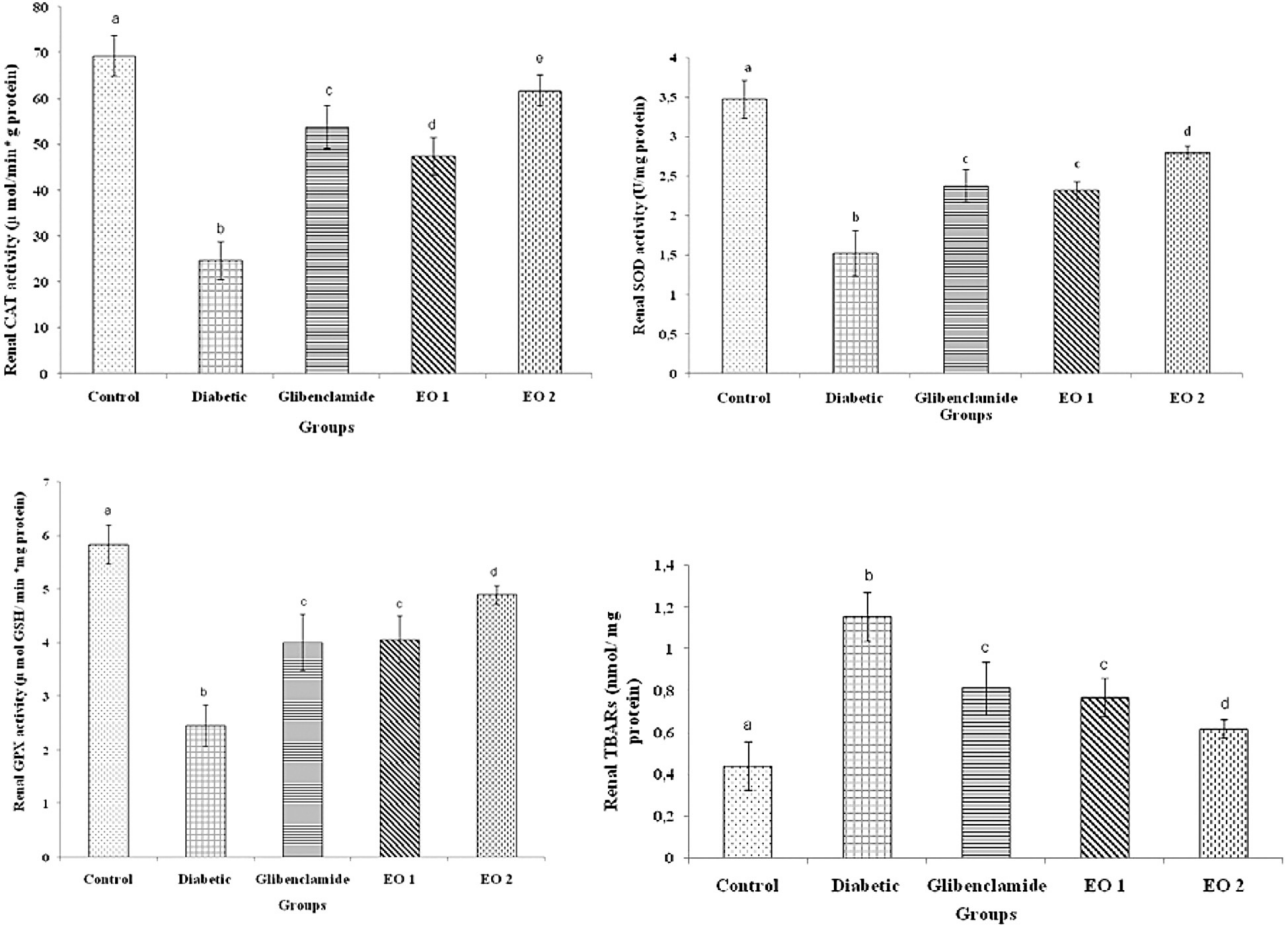
**Effects of leaf essential oil (EO1 and EO2) and Glibenclamide on SOD, CAT, GPX activities and TBARS in kidney of diabetic male wistar rats for 30 days.** Values are expressed as means ± S.E. (n = 8). ^abcd^ The means not sharing a common letter are significantly different among groups (*p* < 0.05).

**Figure 6:**
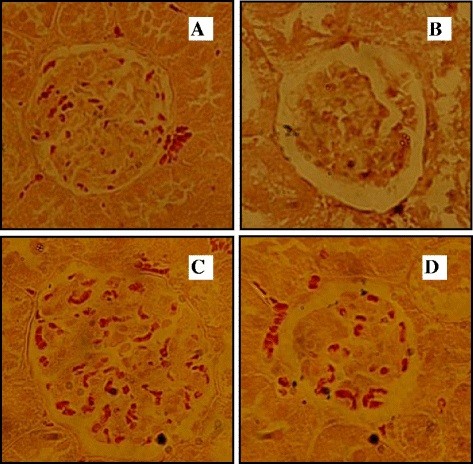
**(A) Normal rat kidney.** (**B**) Diabetic treated rat kidney: tubular epithelial damage messangial capillary proliferation and fatty infiltration. (**C**) Diabetic rat treated with Glibeclemide and (**D**) Diabetic rat treated with essential oil (150 mg/kg): a positive effect was observed (H&E 100×).

In diabetic rats, a decrease in the SOD, CAT and GPX activities by 43.8, 35.7 and 42.12%, respectively, in kidneys was shown at the end of the experiment, Moreover, a significant increase of TBARS in kidneys by 37.39% was obtained in alloxan-treated rats (Figure 5). However, after the essential oil administration, a positive action from nephropathy was clearly observed. In fact the renal SOD, CAT and GPX antioxidant activities increased after the administration of essential oil at 150 mg/kg b.w. Furthermore, the *P. graveolens* oils decreased lipid peroxidation content. Moreover, a normal histological structure was observed (Figure 6A). This positive effect of these fractions was confirmed by histological findings. As shown in Figure 6B, although fatty infiltration was found in the kidneys of diabetic rats, the structure of the glomeruli remained intact in the kidneys of the rats treated with essential oil (Figure 6C, Figure 6D), which is very near to the control rat kidney histological organisation.

## Discussion

Diabetes mellitus, a leading metabolic disorder worldwide, is characterized by hyperglycemia associated with impairment in insulin secretion and/or insulin action as well as alteration in intermediary metabolism of carbohydrate, protein and lipids. Reactive oxygen species play a relevant role in the etiology and pathogenesis of diabetes mellitus and its complications. Lipid peroxide mediated tissue damage has been demonstrated in insulin dependent and non-insulin-dependent diabetes mellitus [15]. As a strategy to counteract the negative effect of oxidative stress, antioxidant-based therapy is promising to minimize the complications in diabetes mellitus. The present study was undertaken to evaluate the hypoglycemic and antioxidant status of leaf essential oil of *P. graveolens* on alloxan induced diabetes. To the best of our knowledge, this is the first report of hypoglycemic activity of this plant. Our results have proved that the leaf essential oil at two different doses, 75 and 150 mg/kg b.w., had significant hypoglycemic and antioxidant effects comparable to those of glibenclamide. It has been reported that glibenclamide produces the hypoglycemic effect by stimulating insulin secretion from β cells of pancreatic islets [16,17]. In the present study, the oral administration of the essential oil of *P. graveolens* brought about significant hypoglycemic effects in alloxan-induced diabetic rats, mainly at 150 mg/kg b.w. The eventual mechanism responsible for the hypoglycemic effect of this plant may result from a potentiation of glucose-induced insulin release or increased peripheral uptake of glucose [18]. In fact, the hepatic glycogen levels in diabetic rats receiving essential oil were significantly higher than those of the normal as well as diabetic rats. The hypoglycemic effect of *P. graveolens* essential oil extract is probably related to its content of several bioactive antidiabetic principles. In addition, the antidiabetic effect of monoterpenes which were the major compounds identified in *Satureja khozestanica* essential oil, was previously demonstrated by Tavafi et al. [16].

Several studies have illustrated the increased lipid peroxidation in clinical and experimental diabetes. It has been established that diabetes leads to increased production of oxygen free radicals [19,20] which exert their cytotoxic effect by causing lipid peroxidation, resulting in the formation of TBARS. In our study, alloxan-induced diabetic rats showed a significant rise in liver and kidney TBARS levels. The treatment of diabetic rats with the leaf essential oil of *P. graveolens* has confirmed significant reduction of TBARS in all analyzed tissues. These data suggest that rats treated with *P. graveolens* oil are less susceptible to peroxidative damage under the challenge of oxidative stress such as diabetes.

It has also been reported that oxidative stress may constitute the key and common event in the pathogenesis of different diabetic complications [21]. This stress results from an imbalance between the production of free radicals and the effectiveness of the antioxidant defence system [22]. Dietary monoterpenes appear to have physiological antioxidant properties, which quench reactive oxygen and nitrogen species, thereby potentially contributing against the pathogenesis of diabetic complications [16,23]. In the present study, we have observed decreased activities of antioxidant enzymes CAT, SOD and GPX in the liver and kidneys of diabetic group. Our results are in harmony with those obtained in previous studies, which suggests that hyperglycemia induces a depletion of the antioxidant system due to the increased lipid peroxidation and formation of free radicals [24-26]. The treatment of alloxan-induced diabetic rats with the leaf essential oil of rose-scented geranium was confirmed to increase the SOD, CAT and GPX activities. The increase may be due to the activation of enzymes by *P. graveolens* essential oil, resulting in a lower superoxide anion level. Besides, the higher CAT, SOD and GPX activity could lead to a reduced reactive oxygen species level in *P. graveolens* oil supplemented group. These results suggest that *P. graveolens* oils reduce oxidative stress by preventing the generation of free radicals and, thus inhibiting the development of diabetes. The reduced oxidative stress and lipid peroxidation observed in the geranium essential oils treated animals may be attributed to the important role of the essential oil as antioxidants. This power may be attributed to their ability to decompose free radicals by quenching reactive oxygen species and trapping radicals before reaching their cellular targets [27]. The antioxidant activity of geranium essential oil could also be assigned to the presence of monoterpenes. The geraniol and β-citronellol were the major monoterpenes detected in the chemical composition of geranium oils (Table 1). Indeed, Singh et al. [28] assessed the antioxidant and radical scavenging activity of two major monoterpenes (citronellal and β-citronellol) present in the essential oil of *Artemisia scoparia*. Moreover, the measured antioxidant activities could be due to the synergistic effects of two or more compounds present in the oils. In this context, Lu and Foo and Nivitabishekam et al. [29,30] reported that most natural antioxidative compounds often work synergistically with each other to produce a broad spectrum of antioxidative properties that create an effective defence system against free radicals.

In Conclusion, the present study clearly demonstrates that the essential oil of *Pelargonium graveolens* not only exhibit a pronounced hypoglycemic effect but also reduce the lipid peroxidation process as well as enhance the antioxidant defence system. For further studies, isolation and investigation of the chemical constituent of the essential oil of *P. graveolens* responsible for the hypoglycemic effect should be undertaken in order to confirm and clarify the mechanism behind this activity.

## Materials and methods

### Chemicals and reagents

Alloxan, catalase, superoxide dismutase (SOD) were purchased from Sigma-Aldrich Chemicals Pvt. Ltd., Bangalore. Serum and hepatic glucose levels were measured using commercial kits from Sigma-Aldrich (Munich, Germany). BCA protein kit was procured from Thermo Fisher Scientific Inc., MA, USA. All other chemicals and reagents used in this study were of analytical grade.

### Plant material

Fresh leaves of *P. graveolens* at flowering stage were collected in March 2008 in Sfax, South-East of Tunisia (34° 47′ 57.04″ N, 10° 45′ 57.89″ E, Altitude 20 m), which is characterized by its arid climate having a mean rainfall of 200 mm per year. The leaves were manually separated and dried in the shade. Then the botanical identification of *P. graveolens* was conducted by Professor Makki Boukhris, botanist in the Faculty of Science of Sfax, Tunisia. Voucher specimens were deposited at the laboratory of Environmental bioprocesses (PG 22), in the Centre of Biotechnology of Sfax.

### Essential oil isolation

Fresh leaves of *P. graveolens* (600 g) were separately grounded and submitted to hydrodistillation for 4 h using a clevenger-type apparatus and the obtained oil was dried over anhydrous sodium sulfate. The essential oil from leaves has a yield of 0.19% on a dry basis.

### Gas chromatography/mass spectrometry (GC/MS) analysis

The gas chromatography/mass spectrometry (GC/MS) analysis of the essential oil was performed using Agilent 6890 N Network gas chromatographic (GC) system equipped with a flame ionization detector. In fact, an aliquot of 1 μl of the essential oil was injected splitlessly into the GC/MS. The samples were diffused in a 30 m-long 0.25 mm i.d. and 0.25 μm-thick DB-5MS column (Agilent Technologies, J&W Scientific Products, USA) and the carrier gas used was helium. The GC oven temperature started at 100 °C and held for 1 min at 260 °C and then for 10 min with 4 °C min^−1^ program rate. While the injector and detector temperatures were set at 250 and 230 °C, respectively, the mass range was scanned from 50 to 550 amu. As for the control of the GC/MS system and the data peak processing, they were conducted by means of MSDCHEM software. With regard to the identification of components, it was realized via the comparison of the retention indices (RI) pertaining to C9–C26 *n*-alkanes and MS corresponding database (Wiley and NIST library) with the mass spectral literature.

### Animals

Male Wistar rats with body weights of 220 ± 10 g, were purchased from the Central Pharmacy (SIPHAT, Tunisia). They were fed on a pellet diet (SICO, Sfax, Tunisia) and water ad libitum. They were also maintained in a controlled environment under standard conditions of temperature and humidity with an alternating light-and-dark cycle (12 h day/12 h night). According to the European convention for the protection of vertebrate animals used for experimental and other scientific purposes (Council of Europe N^o^ 123, Strasbourg, 1985) and the ethics committee for research on laboratory animal use of our institution, rats rearing and experiments of this study were approved.

### Experimental induction of diabetes and treatments

The method of diabetes induction in rats by alloxan was the same as described by Ananthan et al. [14]. Actually, rats were single injected intraperitoneally with a freshly prepared solution of alloxan monohydrate in normal saline at a dose of 150 mg/kg b.w., freshly dissolved in NaCl 0.9% buffer (pH 7). Since alloxan is capable of producing fatal hypoglycemia because of massive pancreatic insulin release, rats were orally treated with 20% glucose solution 6 h after the injections. The rats were then kept for the next 24 h on 5% glucose solution bottles in their cages to prevent hypoglycemia. After 2 weeks, rats with blood glucose level of above (10 mmol /dm^3^) were considered to be diabetic and used for the studies. The rats were randomly divided into five experimental groups (n = 8). Group 1 (C): normal rats. Group 2 (D): diabetic rats (glycemia was more than 10 mmol /dm^3^). Groups 3: diabetic rats treated with Glibenclamide (600 μg/kg.b.w.). Groups 4–5 (EO1 and EO2): diabetic rats orally receiving daily leaf essential oil of *P. graveolens* at 75 mg/ kg and 150 mg/kg respectively.

After one month of treatment, the animals were sacrificed by decapitation, and the trunk blood was collected. Then, the serum was prepared by centrifugation (1500 × g, 15 min, 4 °C). Next, the kidneys and liver were removed, cleaned of fat and stored at −80 °C until future use. Immediately, pieces of liver and kidney were fixed in a Bouin solution for histological studies.

### Analytical methods

The activity of superoxide dismutase was examined by the spectrophotometric method of Marklund and Marklund [31]. Concerning the catalase (CAT), it was assayed colorimetrically at 240 nm and expressed as moles of H_2_O_2_ consumed per minute per milligram of protein, as described by Regoli and Principato [32]. As for the glutathione peroxidise (GPX) activity, it was measured by the method described by Pagila and Valentine [33]. With regard to the lipid peroxidation in the liver and kidneys of control and all treated groups, it was measured by the quantification of thiobarbituric acid-reactive substances (TBARS) determined by the method of Park et al. [34]. Regarding the level of total protein, it was determined referring to Lowry et al. [35] using bovine serum albumin as the standard at 660 nm. Furthermore, serum and hepatic glucose levels were measured using commercial kits from Sigma Munich (Munich, Germany). The Fasting blood glucose was estimated by commercially available glucose strips (Accu-Chek Active) using One Touch Glucometer (Johnson-Johnson). For histological studies, pieces of liver and kidney were fixed in a Bouin solution for 24 h, and then embedded in paraffin. 5 μm-thick sections were stained with hematoxylin-eosin and examined under the Olympus DP-70 light microscope.

### Statistical analysis

All data are presented as the mean ± SE values. The data were evaluated by a one-way analysis of variance using SPSS (Chicago, IL) software and differences between the means were determined using Student’s t-test. Values were considered statistically significant when *p* < 0.05.

## Competing interests

The authors declare that they have no competing interests.

## Authors’ contributions

MB1 prepared the study design, carried out all the biological studies, analyzed and discussion of the data, and drafted the manuscript. MB2 helped with chemical analysis of the essential oil and correction of the manuscript. IF and HJ carried out some biological assays and helped with the manuscript preparation. AF and SS participated in the study design, discussion the data and helped to draft and correction of the manuscript. All authors have read and approved the final manuscript.
